# Prevalence and Intensity of *Perkinsus* sp. Infection in *Mizuhopecten yessoensis* and Its Impact on the Immune Status of Bivalves

**DOI:** 10.3390/pathogens14121303

**Published:** 2025-12-18

**Authors:** Elizaveta Tsoy, Ayna Tumas, Mariia Mokrina, Andrei Grinchenko, Vadim Kumeiko, Daria Lanskikh, Yulia Sokolnikova

**Affiliations:** 1 A.V. Zhirmunsky National Scientific Center of Marine Biology, Far Eastern Branch of Russian Academy of Sciences, 690041 Vladivostok, Russia; lizasun03@mail.ru (E.T.); grishagrin@mail.ru (A.G.);; 2 School of Medicine and Life Sciences, Far Eastern Federal University, 690922 Vladivostok, Russia

**Keywords:** aquaculture, bivalves, scallops, immunity, *Perkinsus*

## Abstract

Despite the economic importance of *Mizuhopecten yessoensis*, little is known about their parasites and immunity. This study, the first to examine the prevalence and intensity of *Perkinsus* across three age groups of scallops from four locations in the Sea of Japan, revealed that the gills, mantle, and digestive glands of one-year-old specimens from mariculture farms are heavily colonized. The cases of infection were notably higher in older specimens (mostly hemolymph and shell), suggesting that they act as carriers of *Perkinsus*. An immunological analysis indicated that when the pathogen is found only in the hemolymph and mantle, there is an increase in plasma protein concentrations, which likely plays a crucial role in resisting infection. However, when hypnospores were present in the mantle and gills, a decrease in reactive oxygen species and granulocytes occurred, accompanied by an increase in hemoblasts and agranulocytes. Phagocytic activity increased only when the pathogen appeared in the digestive gland. This evidence highlights the heightened vulnerability of young scallops, emphasizing the necessity for preventive measures against infection. The current troubling epidemiological situation regarding scallop diseases in the region suggests a rise in epizootics, raising doubts about the sustainability of the scallop farming industry unless timely interventions are implemented.

## 1. Introduction

According to the World Organization for Animal Health (WOAH), some of the most dangerous pathogens affecting commercial bivalve mollusks are protozoans from the genus *Perkinsus*. These microorganisms can cause perkinsosis (also known as dermocystidiosis or Dermo disease), which can lead to 100% mortality in affected populations worldwide [1,2]. To date, seven species of *Perkinsus* have been identified and described: *Perkinsus marinus*, *Perkinsus olseni (atlanticus)*, *Perkinsus qugwadi*, *Perkinsus chesapeaki (andrewsi)*, *Perkinsus mediterraneus, Perkinsus honshuensis*, and *Perkinsus beihaiensis*. The specific pathologies associated with perkinsosis vary depending on both the species of *Perkinsus* and the host organism. These protozoans are found from the east coast of the United States to the waters of Japan and Korea [3], primarily affecting species such as *Crassostrea virginica*, *Mytilus galloprovincialis*, *Mytilus chilensis*, *Mya arenaria*, *Ostrea edulis*, *Ruditapes philippinarum*, *Macoma balthica*, and *Crassostrea hongkongensis* [4,5,6]. Although numerous new species of infected mollusks from new regions are reported each year, the impact of these pathogens on oysters has been the most extensively studied. There is limited data concerning the diseases affecting other bivalve species, particularly economically valuable ones like scallops [7,8,9]. The global scallop farming market is currently valued at approximately US$18.5 billion (3.88 million tons in 2024) and is projected to continue growing. According to FAO data, the main producing countries of *Patinopecten (Mizuhopecten) yessoensis* are Japan, China, South Korea, the Russian Federation, Argentina, Canada, and France.

The first documented cases of epizootics caused by *P. qugwadi* in *M. yessoensis* occurred in 1988 in British Columbia, Canada [10]. Local scallop species, *Chlamys rubida* and *Chlamys hastata*, showed resistance to the infection. After 1995, the prevalence of infection declined sharply, and the last recorded case was in 1997. This led to speculation that the scallop population in Canada had either developed resistance to the disease or that *P. qugwadi* had vanished entirely. However, a 2011 study revealed that *P. qugwadi* infections and the associated mortality still affected scallops in at least one region of British Columbia [10]. In recent years, numerous mariculture enterprises in the Primorsky Territory have also faced mass die-offs of *M. yessoensis* [11]. The first infected scallops were likely imported from Japan to the bays of Primorsky Territory in 1979 (however, there is no information in the literature on cases of scallop infection in countries of the Asia-Pacific region) [12,13]. An analysis from that time showed that 86% of the scallops examined tested positive for trophozoites and prezoosporangia of *Perkinsus* sp. [11]. New cases of *Perkinsus* infection were recorded only in 2014 [14]. Subsequent mass mortality of scallops in the region was noted between 2018 and 2021, particularly after the importation of juveniles from China for cage farming in the Moryak–Rybalov Bay area [15] and Voevoda Bay [14]. It was later discovered that abnormal mortality rates among scallops in cage farming are not uncommon in Primorsky Territory, but aquaculture farms tend not to publicize this issue. For instance, at one enterprise, scallop harvests in the early stages of cage farming yielded up to 3 tons per commercial cycle. Over the years, this figure dropped to 2 tons, then to 600 kg, and recently, some orders harvested less than 100 kg [16]. The current epidemiological situation indicates an increase in the frequency of epizootics, likely due to the rapid development of aquaculture in the Far East and the high adaptive potential of the pathogen. This raises significant questions about the future viability of the scallop farming industry in Primorsky Territory.

Despite the commercial significance of scallops, they have been poorly studied in terms of both parasitology and physiology, especially when compared to mussels and oysters, which have longer cultivation periods and broader research coverage. There is even less information available about the infection of scallops with the protozoan *Perkinsus* in Primorsky Territory, with existing data primarily focused on the frequency of infection [14,16]. The physiological characteristics of the pathogen, its ways of entry and spreading in *M. yessoensis*, as well as the symptoms, progression of the disease, and complications it causes in these mollusks, remain largely uncharacterized [11,14,15]. Therefore, the aim of this study is to assess the prevalence of parasitic protozoa of the genus *Perkinsus* in *M. yessoensis* bivalves in the waters of Peter the Great Bay, Sea of Japan, and to investigate their impact on the host’s immune response.

## 2. Materials and Methods

### 2.1. Animals

**Figure 1:**
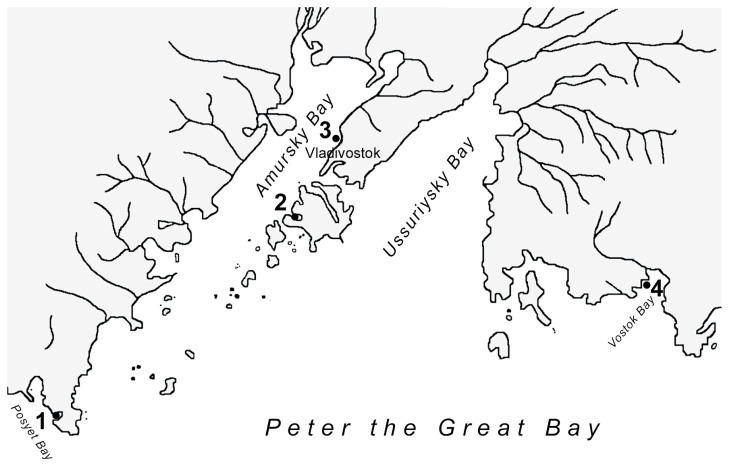
Locations of sampling stations in the Peter the Great Bay of the Sea of Japan: (1) Vityaz Bay, (2) Voevoda Bay, (3) Sportivnaya Gavan Bay, (4) Vostok Bay (near the biological station «Vostok»).

During the expedition conducted in May 2024, samples of organs and tissues from the *M. yessoensis* were collected from four locations: Vostok Bay (n = 24), Sportivnaya Gavan Bay (n = 30), Vityaz Bay (n = 36), and Voevoda Bay (n = 35) in Peter the Great Bay, Sea of Japan. In February 2025, additional samples were collected from Vityaz Bay (n = 60) (Figure 1). The age of the mollusks ranged from 0 to 4 years, determined by measuring the height of the shell with a caliper having an accuracy of 0.1 mm, as per the literature [17]. The shell height of individuals from the “young” age group in Vityaz Bay ranged from 4.62 ± 0.32 cm to 4.44 ± 0.37 cm in Voevoda Bay. For the “middle” age group in Vityaz Bay, the measurements were 7.35 ± 0.35 cm and 7.49 ± 0.45 cm. The “adult” age group in Vityaz Bay had shell heights of 11.22 ± 0.32 cm and 11.47 ± 0.28 cm. In contrast, shell height varied between 9.27 ± 1.65 cm in Sportivnaya Gavan Bay and 8.94 ± 2.37 cm in Vostok Bay.

The collection point near the Vostok biological station in Vostok Bay is considered non-impacted. This area of the bay is more open and is influenced by stronger southern and eastern winds and river currents. It features specific hydrodynamic processes that help promote the dispersion of pollutants. Additionally, part of Vostok Bay is protected by the State Natural Complex of the Vostok Bay Marine Reserve [18,19,20]. On the other hand, Sportivnaya Gavan is situated near the Vladivostok urban agglomeration [21,22] and experiences high to moderate levels of pollution [23,24]. An increase in cases of intensive microalgae growth has been noted here, indicating eutrophication. Pollution in this water area primarily results from the seaport and various types of wastewaters [25]. Vityaz Bay and Voevoda Bay, however, are not affected by untreated wastewater or other sources of pollution, despite recreational activity during the summer. As a result, these areas are considered background sites. Additionally, mixed aquaculture is practiced in these bays [26,27].

### 2.2. Hemolymph Collection and Processing

Hemolymph was collected from the posterior adductor muscle sinus using a 5 mL syringe. To prevent hemocyte aggregation, 1 mL of the collected hemolymph was placed in microtubes that had been pre-chilled in an ice bath. An aliquot of the native hemolymph (200 μL) was fixed with 8% paraformaldehyde (PFA; Sigma-Aldrich, Burlington, USA) to evaluate the degree of *Perkinsus* contamination. The remaining hemolymph was centrifuged at 800× *g* for 12 min at 15 °C. The obtained supernatant was transferred into cryovials and frozen at −85 °C for further analysis of humoral parameters. The hemocyte suspension was then resuspended in an artificial sea water (ASW) containing 460 mM NaCl, 9.4 mM KCl, 48.3 mM MgCl_2_ · 6H_2_O, 6 mM NaHCO_3_, 10.8 mM CaCl_2_ · 2H_2_O and 10 mM N-(2-hydroxyethyl) piperazine-N’-2-ethanesulfonic acid (HEPES). This medium had an osmolality of 1090 mOsm and a pH of 7.5, and it was used to assess cellular immunity parameters, including total and differential hemocyte counts, phagocytic activity, and the content of reactive oxygen species.

### 2.3. Assessment of the Degree of Contamination of Mollusks

To assess the level of infection in *Perkinsus* mollusks, necropsies were conducted, and both the bodies and shells were meticulously examined for abnormalities. This included checking for shell deformations, erosions, watery tissues, and the presence of pustules in the organs.

Subsequently, samples measuring 1 cm^3^ were collected from the digestive gland, gills, and mantle, along with a swab from the outer surface of both shell valves of animals from Voevoda Bay and Vityaz Bay. These samples were then treated with 5 mL of 2 M NaOH and incubated at 60 °C for 6 h to lyse immature trophozoites and host tissue. After incubation, the samples and fixed hemolymph were washed three times with bidistilled water using centrifugation at 1000× *g* for 10 min. The supernatant was carefully removed, and a 50 µL aliquot of the resulting precipitate was transferred to separate microtubes, where Lugol’s iodine solution (at a 1:1 ratio) was added. After 10 min, the number of prezoosporangia, which stained black or dark blue, was counted using a hemacytometer under an Olympus IX73 microscope (Olympus, Tokyo, Japan).

### 2.4. Evaluation of Immune Parameters of Hemolymph

To obtain a transient hemocyte cell culture, 45 µL of the cell suspension was added to both black and transparent 96-well flat-bottomed plates in triplicate. The plates were incubated in a humidified chamber for 20 min to promote cell adhesion to the substrate. Bacteria of the genus *Staphylococcus* sp. were used as biotic particles for the in vitro phagocytosis reaction. The bacteria were heat-inactivated at 72 °C for 1 h and labeled with a 0.1% fluorescein-5-isothiocyanate (FITC, MP Biomedicals, Santa Ana, CA, USA) solution, which was prepared in 0.1 M carbonate-bicarbonate buffer (pH 9.3). These labeled bacteria were added to the hemocytes at a concentration of 15–20 cells per hemocyte, corresponding to 60 million CFU/mL. One hour after the bacterial addition, a 0.1% trypan blue solution was added to quench the fluorescence of any non-internalized bacteria. After 5 min, the cells were washed three times with ASW and then fixed with 4% paraformaldehyde for 1 h. The phagocytic activity was subsequently analyzed using a Cytation 5 Imaging Reader (BioTek Instruments, Winooski, VT, USA) with excitation at 485 nm and emission at 535 nm. A spontaneous nitroblue tetrazolium (NBT; Sigma-Aldrich, Burlington, NJ, USA) assay was performed to detect reactive oxygen species in hemocytes. After the cells adhered to the plate, a 0.004% NBT solution prepared in ASW was added for 30 min. Following this, a solution containing 120 µL of 2 M KOH and 140 µL of dimethyl sulfoxide was added to the wells to solubilize the dye. The plate was then shaken and analyzed using a Bio-Rad xMark Microplate Absorbance Spectrophotometer (Bio-Rad Laboratories, Hercules, CA, USA) at a wavelength of 630 nm.

To count the total and differential numbers of circulating hemocytes, 500 µL of the cell suspension was fixed with 8% PFA. Immediately before analysis, the cells were washed out of the PFA using ASW, followed by centrifugation for 12 min at 800× *g*. Samples were analyzed using a CytoFLEX flow cytometer (Beckman Coulter, Indianapolis, IN, USA). At least 10,000 events (individual signals from cells and other particles in the suspension) were analyzed for each sample. Two-parameter histograms of cell size and granularity distribution were constructed by analyzing forward scattering channel (FSC) and side scattering channel (SSC) signals.

Total plasma protein concentration measurement was performed in 96-well UV-transparent microplates (200 µL of sample per well) using a Bio-Rad xMark Microplate Absorbance Spectrophotometer (Bio-Rad Laboratories, Hercules, CA, USA) with default settings for the UV method.

### 2.5. Data Analysis

Statistical analysis was performed using Microsoft Excel 2010 and Statistica 6.0. The Kolmogorov–Smirnov test showed that the data distributions did not follow the normal distribution, so the analysis was carried out using nonparametric statistics. The Mann–Whitney U rank test was used to assess differences in parameters between the studied water areas. Correlation analysis was performed using the Spearman correlation coefficient (R). In all analyses, significance levels were set at *p* < 0.05. The results of the study are presented as mean values ± confidence intervals (CI).

## 3. Results

**Figure 2:**
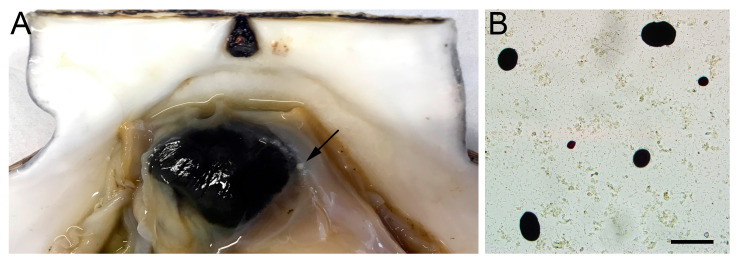
*M. yessoensis* infected with *Perkinsus*. The black arrow indicates the location of the white pustule (**A**). *Perkinsus* prezoosporangia stained with Lugol’s iodine (**B**). Scale bar: 30 µm.

An external examination of the mollusks’ bodies and shells showed no individuals with obvious signs of *Perkinsus* infection, except for a few with white pustules on their mantles and gills (Figure 2A). Microscopic analysis (Figure 2B) of tissue sample suspensions stained with Lugol’s iodine solution revealed that *Perkinsus* hypnospores are round or slightly oval, ranging in size from 6.00 to 26.67 µm. These hypnospores were found in scallops from all examined waters (Table 1).

**Figure 3:**
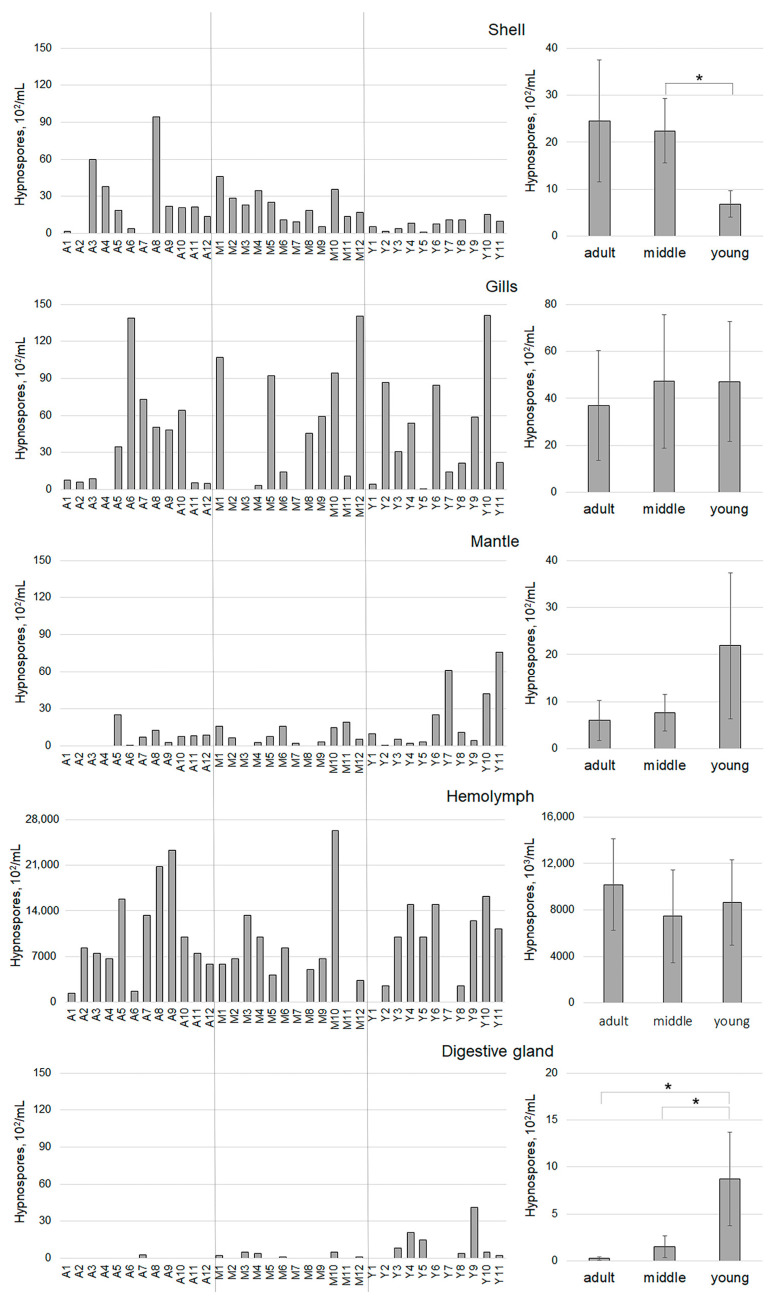
Organ and tissue contamination levels of individuals (**left**) and the average values for each age group (**right**) of *M. yessoensis* caught in Voevoda Bay. Vertical lines separate the age groups: A—adults (3 years and older), M—middle-aged (2 years), Y—young (1 year). Asterisk (*) indicates significant differences (*p* < 0.05).

A contamination study of 35 mollusks collected from Voevoda Bay in 2024, representing three age groups (adult, 3 years and older; middle, 2 years; and younger, 1 year), indicated that the younger specimens were the most infected. The hemolymph, mantle, gills, and digestive gland were the most heavily infected areas (Table 1, Figure 3). Although all specimens studied contained hypnospores in the hemolymph of adult scallops, 81.8% of the 1-year-old group were infected, with 83.3% of the middle-aged group also showing infection. Notably, 100% of the gills in young specimens were contaminated with *Perkinsus*. Interestingly, the highest number of prezoosporangia was found in the middle-aged group (an average of 4735 hypnospores per milliliter), even though this group had the lowest number of infected individuals. On the shell surface, the highest counts of hypnospores were detected in both the adult and middle-aged specimens. Prezoosporangia were also identified in the mantle and digestive gland of scallops across all age groups, but in much smaller quantities compared to the other organs and tissues.

**Figure 4:**
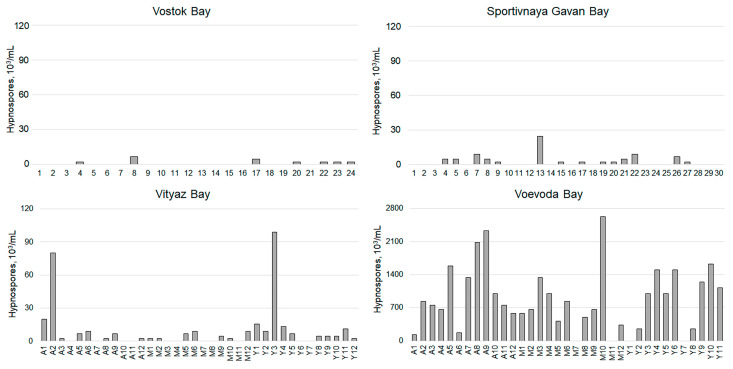
Contamination levels of hemolymph of *M. yessoensis* caught from the waters of Peter the Great Bay, Sea of Japan (A—adult, M—middle-aged, Y—young).

An analysis of the *Perkinsus* contamination in the organs and tissues of *M. yessoensis* scallops collected from Voevoda Bay (Figure 3) and other locations (Figure 4) showed that the presence of hypnospores in the hemolymph (Table 1) is sufficient and informative for assessing this parameter. Consequently, further analysis was conducted only on the hemolymph, which is the most atraumatic method.

Twenty-four individuals were collected from Vostok Bay. An analysis of hemolymph contamination revealed that 26% of these specimens contained hypnospores (Table 1, Figure 4). The maximum count recorded was 5667 hypnospores/mL, with an average of 926 ± 579 hypnospores/mL.

Thirty specimens of *M. yessoensis* were recovered from Sportivnaya Gavan Bay. The proportion of animals infected with *Perkinsus* reached 47%, with a maximum hypnospore count in the hemolymph of 24,444 hypnospores/mL. On average, these animals had 3667 ± 1470 hypnospores.

From Vityaz Bay, 36 specimens of varying ages were collected: 12 individuals aged three years or older (“adult”), 12 aged two years (“middle-aged”), and 12 aged one year (“young”). Compared to the reference area, *Perkinsus* contamination in animals from Vityaz Bay was over ten times higher. The “young” group showed the highest susceptibility to infection, with more than 83.02% infected. Among the “adult” group, 66.67% of the mollusks were infected, and their hypnospores count was similar to that found in the reference waters. Overall, the incidence of *Perkinsus*-infected mollusks in Vityaz Bay was 69.44%.

Thus, the highest infection rates of adult (market-grade) scallops were observed in waters with aquaculture farms, Voevoda Bay (89%) and Vityaz Bay (69.44%), while the lowest rate was found in Vostok Bay (26%). A comparative analysis of *Perkinsus* counts in mollusks from Vityaz Bay collected in May 2024 and February 2025 revealed a significantly higher number of hypnospores and their frequency of occurrence in animals caught during the spring (*p* < 0.05) (Table 1).

**Figure 5:**
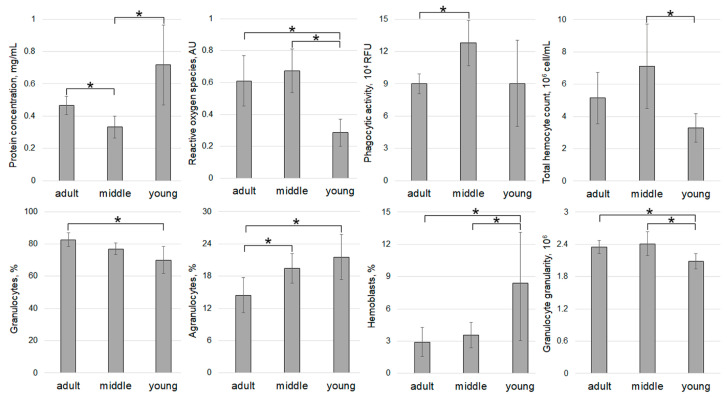
Average values of immunity parameters and the content of different types of hemocytes in the hemolymph of *M. yessoensis* caught in Vityaz Bay. Asterisk (*) indicates significant differences (*p* < 0.05).

**Figure 6:**
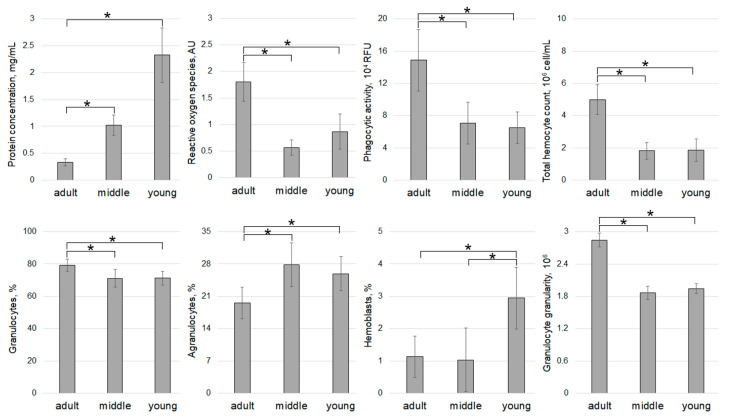
Average values of cellular immunity parameters and the content of different types of hemocytes in the hemolymph of *M. yessoensis* caught in Voevoda Bay. Asterisk (*) indicates significant differences (*p* < 0.05).

Since scallops from Voevoda Bay and Vityaz Bay exhibited the highest hypnospore counts compared to other samples, a more detailed analysis of their immune status based on age was conducted (Figure 5 and Figure 6).

**Figure 7:**
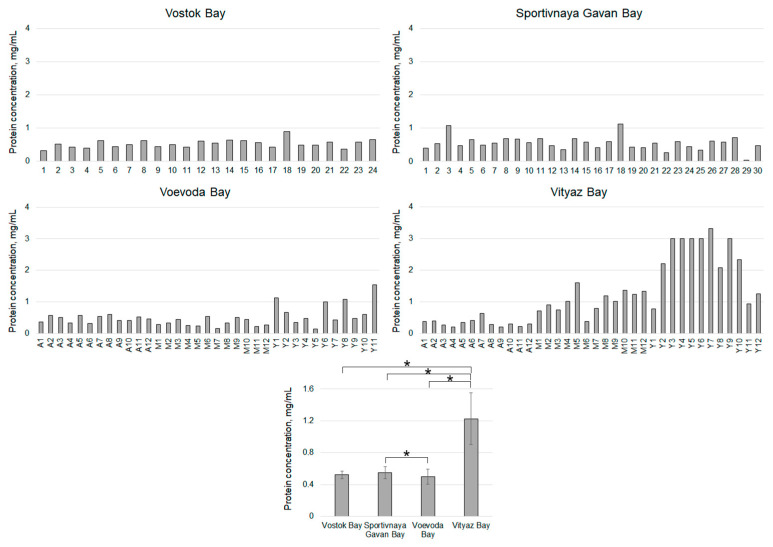
Plasma protein concentration of *M. yessoensis* caught from the waters of Peter the Great Bay, Sea of Japan (A—adult, M—middle-aged, Y—young). Asterisk (*) indicates significant differences (*p* < 0.05).

The analysis of hemolymph parameters in mollusks caught in 2024 revealed the highest plasma protein concentration (PC) (1.23 ± 0.27 mg/mL) in animals from Vityaz Bay (Figure 7). The values showed considerable variation, ranging from 0.20 to 3.32 mg/mL. Specimens in the “young” group had the highest PC (2.33 ± 0.44 mg/mL), while animals aged 3 years and older had approximately seven times lower PC (0.33 ± 0.06 mg/mL) (Figure 5).

Specimens from Sportivnaya Gavan Bay had significantly lower concentrations of PC, which were almost double that of animals from Vityaz Bay (0.55 ± 0.06 mg/mL) (Figure 7). The average PC value (0.52 ± 0.18 mg/mL) for the entire sample of animals from Vostok Bay did not differ significantly from that of animals from Sportivnaya Gavan Bay. The lowest PC (0.50 ± 0.08 mg/mL) was found in mollusks from Voevoda Bay (Figure 6). The highest mean PC values (0.72 ± 0.21 mg/mL) were observed in the “young” age group, with values ranging from 0.14 to 1.54 mg/mL, while the lowest values (0.33 ± 0.06 mg/mL) were seen in middle-aged scallops (ranging from 0.16 to 0.53 mg/mL). The mean PC value in the “adult” group (0.47 ± 0.05 mg/mL) was slightly higher than that of the middle-aged group (Figure 6).

**Figure 8:**
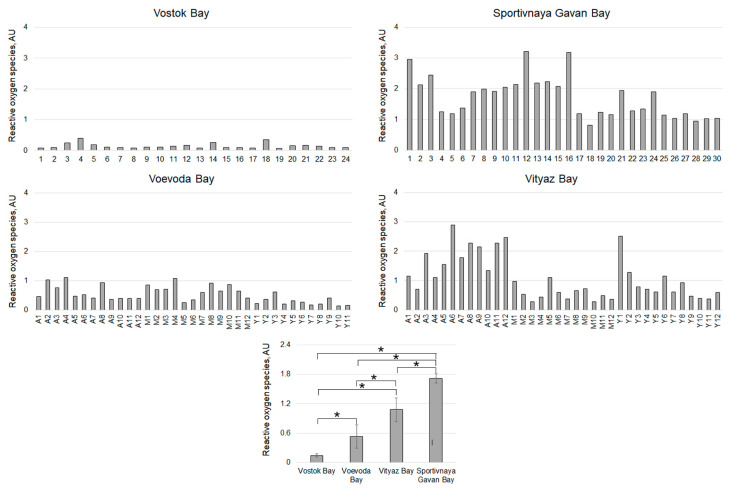
Content of reactive oxygen species in hemocytes of *M. yessoensis* caught from the waters of Peter the Great Bay, Sea of Japan (A—adult, M—middle-aged, Y—young). Asterisk (*) indicates significant differences (*p* < 0.05).

The highest concentration of reactive oxygen species (ROS) in hemocytes was recorded in animals from Sportivnaya Gavan Bay, measuring 1.72 ± 0.20 AU, with a range from 0.82 to 3.20 AU (Figure 8).

In scallops from Vityaz Bay, the oldest individuals exhibited the highest ROS levels at 3.06 AU, averaging 1.82 ± 0.32 AU (Figure 5). Notably, the “young” group demonstrated the widest variation, ranging from 0.38 to 2.52 AU, while the middle-aged group showed the least variability with an average of 0.57 ± 0.12 AU. For the NBT-test, the highest values in scallops from Voevoda Bay were observed in “adult” group (1.12 and 1.03 AU) and middle-aged scallops (1.08 AU). The older animals exhibited the greatest variability in this parameter, with a range from 0.37 to 1.12 AU (Figure 6). Middle-aged scallops had a similar range of NBT values, from 0.26 to 1.08 AU. In contrast, young mollusks had significantly lower NBT values (0.29 ± 0.07 AU), nearly half of those in other age groups (middle group: 0.67 ± 0.12 AU; adult group: 0.61 ± 0.13 AU), and showed little variation (ranging from 0.16 to 0.62 AU). Among the four locations studied, hemocytes from Vostok Bay had the lowest ROS concentration (0.15 ± 0.03 AU), and this indicator also showed minimal variation (from 0.06 to 0.44 AU) (Figure 8).

The most pronounced hemocyte phagocytic activity (PA) was observed in scallops from Voevoda Bay, with an average of 103,129 ± 13,062 RFU, predominantly in middle-aged individuals (127,861 ± 17,523 RFU) (Figure 6 and Figure 8). The mean PA values in the other groups did not differ significantly (adult group: 90,137 ± 7723 RFU; young group: 90,321 ± 33,627 RFU).

**Figure 9:**
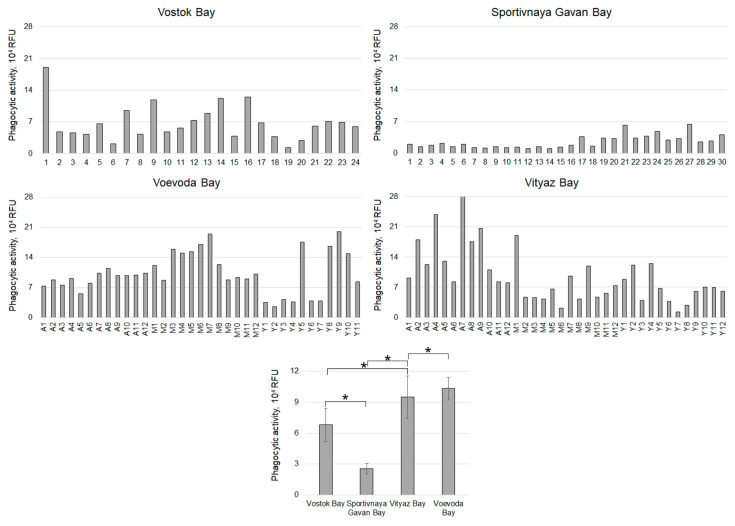
Phagocytic activity of hemocytes of *M. yessoensis* caught from the waters of Peter the Great Bay, Sea of Japan (A—adult, M—middle-aged, Y—young). Asterisk (*) indicates significant differences (*p* < 0.05).

Hemocytes from Vityaz Bay exhibited a similar PA (94,934 ± 17,248 RFU), with values ranging from 12,831 to 281,890 RFU (Figure 5 and Figure 9). The oldest individuals in this group had the highest PA (148,845 ± 32,042 RFU), while the youngest had the lowest (65,105 ± 16,978 RFU). The average PA in specimens from Vostok Bay was approximately 1.5 times lower (67,978 ± 13,309 RFU) than in animals from Vityaz Bay. The lowest PA (25,589 ± 4371 RFU) was recorded in scallops from Sportivnaya Gavan Bay, with values ranging from 10,577 to 63,920 RFU (Figure 9).

**Figure 10:**
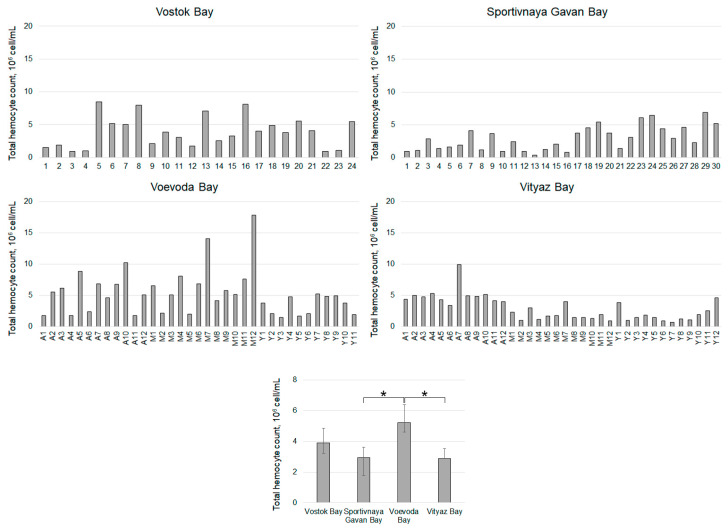
Total hemocyte count in *M. yessoensis* caught from the waters of Peter the Great Bay, Sea of Japan (A—adult, M—middle-aged, Y—young). Asterisk (*) indicates significant differences (*p* < 0.05).

The total hemocyte count (THC) in scallops from the four study areas varied slightly (Figure 10) but was significantly higher in individuals from Voevoda Bay (5.24 ± 0.41 million cells/mL), particularly in the middle-aged group (Figure 6). In contrast, the THC in animals from the reference area, Vostok Bay (3.90 ± 0.33 million cells/mL), was 1.5 times lower. Scallops from Sportivnaya Gavan Bay (2.96 ± 0.23 million cells/mL) (Figure 10) and Vityaz Bay (2.90 ± 0.23 million cells/mL) (Figure 5 and Figure 10) had similar cell counts. In the “adult” group of mollusks from Voevoda Bay, the number of hemocytes practically corresponded to that of animals from Vityaz Bay and varied from 5 to 5.14 million cells/mL.

**Figure 11:**
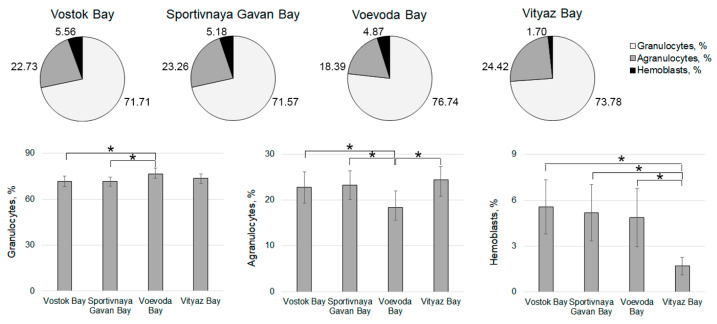
Ratio of hemocyte types in *M. yessoensis* caught from the waters of Peter the Great Bay, Sea of Japan. Asterisk (*) indicates significant differences (*p* < 0.05).

The differential count of hemocytes in *M. yessoensis* collected from the four locations (Figure 11) revealed that granulocytes were the predominant hemocyte type in all areas, constituting 71.57% of the total in Sportivnaya Gavan Bay and 76.74% in Voevoda Bay. Agranulocytes were the second most common type, making up 18.39% in Voevoda Bay and 24.42% in Vityaz Bay. Hemoblasts varied from 1.70% in Vityaz Bay to 5.56% in Vostok Bay, with Vityaz Bay showing a significantly lower hemoblast proportion of just 1.70%. The proportions of granulocytes (73.88%) and agranulocytes (24.42%) in Vityaz Bay were at their highest (Figure 5). A similar distribution pattern of hemocyte types was seen in individuals from Vostok Bay (hemoblasts 5.56%, agranulocytes 22.73%, granulocytes 71.71%) and Sportivnaya Gavan Bay (hemoblasts 5.18%, agranulocytes 23.26%, granulocytes 71.57%). Like those in Vityaz Bay (Figure 5) and Voevoda Bay (Figure 6), adult mollusks had the highest number of granulocytes and the lowest number of agranulocytes and hemoblasts.

A correlation analysis of hemolymph parameters in *M. yessoensis* across all samples from different water sources revealed several key relationships. Notably, the THC exhibited a positive correlation with PA at 0.38, while showing a negative correlation with PC at −0.33. Indicators of cellular functional activity, such as ROS and PA, demonstrated an inverse relationship with PC, with correlation values of −0.13 and −0.28, respectively. Granulocyte and agranulocyte granulation levels were strongly correlated with each other at 0.84, and both types of cells also demonstrated a negative correlation with PA, registering −0.31 for granulocytes and −0.41 for agranulocytes. Additionally, the proportion of hemoblasts showed a negative correlation with PA at −0.22 and with the proportion of granulocytes at −0.48. Among all immune parameters assessed, only PA showed a positive correlation with the number of hypnospores of *Perkinsus* in the hemolymph, with a correlation value of 0.37. In differential analysis by water area, correlations generally followed a similar trend (refer to Table 2, Table 3 and Table 4) but were more pronounced in the sample from Vityaz Bay (Table 5).

In scallops from Voevoda Bay, additional correlation analyses were performed to examine the relationship between tissue and organ contamination and immune parameters across different age groups. In the adult age group, PC increased significantly with higher contamination levels in both the mantle (0.53) and hemolymph (0.64), as well as the digestive gland (0.22). Conversely, in the young age group, this dependence was noted only for the mantle (0.38), while the correlation with the digestive gland was negative at −0.52. In the middle-aged group, a significant correlation (0.71) was observed only with the hemolymph. THC levels increased only in the adult age group with contamination in the hemolymph (0.63), mantle (0.38), gills (0.49), and the digestive gland (0.31). In the middle-aged and young group, only a negative moderate correlation between THC and the presence of hypnospores in the hemolymph was observed. PA levels rose in all specimens regardless of age, with increased contamination in the digestive gland (0.37), with the most significant increase seen in the young age group (0.63). A direct correlation was also found between PA and hemolymph contamination in the young group (0.35). In the adult age group, this correlation extended to the mantle (0.43) in addition to hemolymph (0.33). The NBT test scores across all age groups showed an inverse correlation with contamination in the mantle (−0.46), gills (−0.23), and hemolymph (adult group −0.20; young −0.12), except for the middle age group, which showed a positive correlation (0.46). When infections were detected in the mantle and gills, there was an inverse correlation with the number of granulocytes, ranging from −0.40 to −0.55 across all age groups. This correlation slightly increased in the presence of *Perkinsus* in the hemolymph and digestive gland. The proportion of agranulocytes decreased significantly only in adult scallops (−0.61) but increased in the middle (0.25) and young (0.55) age groups. In the presence of *Perkinsus* in the hemolymph, there was a positive correlation observed in all age groups, ranging from 0.20 to 0.50. A significant change in the number of hemoblasts was noted only when hypnospores were present in the mantle and gills of all mollusk age groups (adult group—0.23; middle—0.37; young—0.43).

For scallops from Vityaz Bay aged three years and older, *Perkinsus* contamination directly correlated with PC (0.37). In the young age group, a stronger correlation was noted with PA (0.61), THC (0.42), and ROS (0.20). In the middle-aged group, contamination correlated only with the content of ROS (0.33).

## 4. Discussion

High mortality rates of bivalve mollusks due to various infestations pose a significant challenge in aquaculture, leading to considerable economic losses in the industry. Currently, mollusks affected by *Perkinsus* are particularly vulnerable, as this parasite has a high pathogenicity and there are limited control measures available [28]. For instance, *Perkinsus* was responsible for 50–80% of the mortality of *Ruditapes decussatus* in the Algarve (southern Portugal) [28], 44% of the mortality of *Crassostrea gasar* in Brazil [29], and up to 40% of the annual cumulative mortality of *Ruditapes philippinarum* in Galicia (northwest Spain) [30]. In Korea, the disease known as perkinosis led to an 80% reduction in the landings of *R. philippinarum* [31]. Additionally, *P. olseni* has caused the collapse of scallop populations in Japan and China [31,32,33].

*M. yessoensis* is ecologically and economically important, but recent years have seen declines in its populations across various farming areas [14,34,35]. Studies conducted in Pacific waters have shown that mass mortality among scallops is also linked to the protozoan *Perkinsus* [14,16,36,37]. *P. qugwadi* was identified in *M. yessoensis*, which was imported to the west coast of Canada from British Columbia for cultivation [36]. This parasite caused an epizootic disease among cultured scallops, resulting in nearly 100% mortality of young individuals (up to 1 year old). Mortality among older individuals (over 2 years) was about 60%. Surviving scallops often exhibited purulent lesions in nearly all their organs [16,37]. While some scallops showed resistance to infection with *P. qugwadi*, the unpredictable emergence of the parasite and the high mortality rates posed a significant threat to the scallop farming industry in region [38]. In Primorsky Territory, the first outbreaks of perkinsosis were reported in late 1979, following the importation of juvenile scallops from Japan. After the mollusks were released, trophozoites and prezoosporangia of *Perkinsus* sp. were found in 86% of the individuals [12]. Further research conducted in 2014 revealed the presence of the pathogen in only 11.1% of scallops in Severnaya Bay, Khasansky District (the same area as Vityaz Bay in our study). By 2016, the proportion of infected individuals had risen to 35%, and in 2021, it increased to 66.7% among mollusks from the nearby Narva Bay. However, the average parasite load per scallop was relatively low, comprising 10–15 hypnospores and approximately 1300 growing and dividing parasite cells [14]. The next outbreak, which occurred in 2018, was also linked to the import of juvenile scallops, this time from China. Initially, natural factors and technological farming processes were considered as possible causes of the animal deaths; however, *Perkinsus* was subsequently detected in the scallop tissues [15]. Our standard microscopic analysis utilizing Rey’s and Lugol’s mediums confirmed the presence of *Perkinsus* prezoosporangia in the tissues and organs of *M. yessoensis*, with sizes ranging from 6 to 27 µm. Literature indicates that the sizes of hypnospores of *P. olseni* range from 10 to 20 µm [39,40], *P. marinus* from 5 to 15 µm [41], *P. chesapeaki* from 25 to 85 µm [42], and *P. beihaiensis* from 35 to 65 µm in oysters [43], 12 to 45 in *Mytilus galloprovincialis*, and from 8 to 27 in *Mytilus coruscus* [44,45]. Furthermore, it has been noted that during cultivation, hypnospores can increase in size from smaller dimensions to 20–70 µm during the incubation period [46].

A comparative analysis of microbial contamination in mollusks from four water areas (Vostok Bay, Sportivnaya Gavan Bay, Voevoda Bay, and Vityaz Bay) revealed the highest number of hypnospores in scallops found in Voevoda and Vityaz Bays. Two independent surveys conducted in 2021 by Boutorina and Degteva, as well as by Gavrilova and colleagues, indicated the highest levels of microbial contamination in scallops from Voevoda Bay, with rates of 100% and 80%, respectively. These contamination levels were notably higher than those found in Vostok Bay, Narva Bay, Rifovaya Bay, and Moryak-Rybolov Bay [11,14]. Voevoda Bay is home to two aquaculture enterprises: Dalstam-Marin LLC and Russkaya Marikultura (Russian Mariculture) LLC. Additionally, the Effektivnaya Energiya (Efficient Energy) eco-farm is located in Vityaz Bay. Recently, these enterprises have faced large-scale epizootics that resulted in the mass mortality of *M. yessoensis* [11,14,15]. From a parasitological perspective, scallops have been studied inadequately due to the narrow scope of their rearing areas. Consequently, increased mortality is often linked to changes in optimal rearing conditions [15,47]. The high stocking densities in mariculture farms [48], along with the proximity of settlements that have anthropogenic influences, likely contribute significantly to the high levels of contamination in these waters. Furthermore, these waters are situated in the inner parts of Posyet Bay (Vityaz Bay) and Amur Bay (Voevoda Bay). While this location is essential for scallop cultivation, it also leads to eutrophication and elevated water temperatures. Seasonal increases in temperature during summer are stressful for mollusks and can promote the proliferation and spread of *Perkinsus*, a phenomenon similarly observed in the oyster *C. virginica* [49]. Anthropogenic pollution also lowers the resistance of scallops to parasites; factors such as shipping and coastal runoff can result in the accumulation of heavy metals and organic pollutants in the water and bottom sediments. Previous studies have demonstrated that chemical pollution acts as an immune suppressor in shellfish, reducing hemocyte PA and overall immune defense efficiency, while increasing susceptibility to parasitic infections, including *Perkinsus* [2,4,50,51]. However, we believe that the primary factor leading to high parasite prevalence is still the dense stocking of cultured scallops in cages. Direct transmission of zoospores and trophozoites from infected individuals to healthy ones occurs, significantly accelerating the spread of disease. This has led to outbreaks of epizootics in aquaculture farms across the USA, Europe, and Australia [52,53,54]. Among infected shellfish, the prevalence of infection has ranged from 23 to 100%. A survey in 2001 indicated that the intensity and prevalence of the pathogen in *R. philippinarum* were much higher in individuals from marine farms compared to those in natural conditions [31].

Several authors have reported that the lowest bacterial loads on shellfish and the mildest severity of perkinsosis are observed in early spring, whereas late autumn tends to see the highest rates of infection and mortality peaks [55]. It has been reported that oyster mortality due to the pathogen *P. marinus* significantly decreases when temperatures drop to 20 °C. Besides temperature, water salinity plays a crucial role in disease prevalence. *P. marinus* does not survive at salinity levels below 3 ‰ and thrives best at 29–35 ‰ [7]. For *P. olseni*, the optimal temperature and salinity ranges are 19–28 °C and 25–35 ‰, respectively. The maximum reproduction of *P. honshuensis* trophozoites occurs at 28 °C and salinity levels of 18–21 ‰ [50]. Similarly, temperature and salinity significantly impact the development of prezoosporangia in *P. chesapeaki*. Hypnospores isolated in winter can release motile zoospores at 10 °C, although their development rate is considerably lower than at 20 °C and 30 °C. The optimal salinity range for this species is between 15 and 35 ‰ [50]. In the scallops we studied, the proportion of infected individuals and the degree of infection in their hemolymph were significantly higher in spring compared to winter. Additionally, it is well known that the gonadal cycle significantly influences the immune system of mollusks. Since scallops spawn in May [48], which is when we collected our samples, it can be hypothesized that this cycle also affected the pathogenicity of *Perkinsus*.

In addition, many authors indicate a positive correlation between the age of the host and the intensity of *Perkinsus* infection, meaning that older hosts tend to have stronger infections [4]. In our observations, the incidence of infected individuals was highest among those aged 3 years and older, reaching 100% in some cases. Interestingly, while the most contaminated animals were younger (1 year old), this finding aligns with data from studies by Casas and colleagues [12,37,56] and Gavrilova and colleagues, who examined scallops aged 0–2 years [11]. These authors noted that mollusks in their first year of life exhibit damage and detachment of soft tissue from the shell, and that 1–2-year-old animals show blackening and curvature of the shell. However, we did not observe similar symptoms. It has been shown that once the protozoan *Perkinsus* enters the mucous mantle secretion, it produces a complex of factors that mask it and induce the capture of the pathogen by pallial hemocytes [56]. This facilitates its penetration into the host organism [57,58]. *Perkinsus* can invade the mollusk through mantle organs, such as labial palps, gills, and the mantle itself, by binding to hemocyte receptors, such as the galectin CvGal [58]. Once captured by the hemocytes and entering the hemal system, the pathogen begins to reproduce actively and continue its expansion [59]. Primary infection foci can arise in various locations, but they are predominantly found in the epithelium of the stomach and digestive gland, as well as in the gills. As the disease progresses, notable symptoms include a decrease in hemocyte count (resulting in parasitic anemia), an increase in the number of free sporangia and trophozoites in the sinuses, extensive inflammation [51], and multifocal encapsulation reactions in connective tissues. Other symptoms include blanching, hydration, hemocytosis, tissue lysis, and severe exhaustion of the infected organism [59,60,61]. When infecting a new host, zoospores attach to the shell or mucosa of the mollusk and subsequently penetrate its tissues and organs. Infected scallops also display a taut mantle with a weak response to touch, and the muscle may easily separate from the shell. Single or multiple creamy-white pustules, up to 5 mm in diameter, typically appear on the organs, particularly the gonads, digestive gland, and mantle [38], which we also observed in isolated cases. Burreson and colleagues noted significant variations in the response to *P. chesapeaki* infection among different mollusk species [42]. In *Mya arenaria* and *Tagelus plebeius*, extensive encapsulation of *P. chesapeaki* cells by hemocytes was typically observed, often with the parasite cells laminated within an eosinophilic acellular matrix [42]. In contrast, *Macoma balthica* did not show parasite cells, but there was heavy hemocytic infiltration of infected tissues and active phagocytosis of the parasite cells [42]. Oysters infected with *P. marinus* exhibited emaciation, decreased growth rates, and more transparent, watery mantle tissues [60,61]. Additionally, *Perkinsus* infection in mollusks has been associated with infiltration of connective tissue, digestive system epithelium, and mantle by hemocytes and pathogen cells, as well as necrosis of digestive gland epithelial cells and tissue destruction in the gill filaments and gonads [51,60,62].

Our examination of the mollusks revealed the presence of hypnospores in all examined tissues (mantle, gills, digestive gland, hemolymph, and shell). The highest concentration was found in the hemolymph, which is the primary site for pathogen dissemination [28]. In some individuals, we also detected small numbers of prezoosporangia in the digestive gland, a sign that the disease may have reached its terminal stage [54,56,63]. Similarly to the digestive gland, the mantle was the least contaminated area. Among the three age groups studied, the young group had the highest levels of contamination, with their hemolymph, mantle, gills, and digestive gland being most intensively infected. Thus, it can be concluded that *Perkinsus* is capable of adhering to the shells of mollusks regardless of age. However, due to the highest shell contamination found in adult and middle-aged scallops, these are likely the primary carriers of the pathogen, transmitting it to younger generations year after year. The elevated concentration of hypnospores in the hemolymph of adult mollusks, paired with their near absence in the digestive gland, suggests that these adult specimens may possess a more developed immune system, allowing them to resist the pathogen more effectively than younger specimens.

The primary defense system responsible for immunity in bivalves, such as scallops, is the hemolymph. This fluid consists of various types of hemocytes (immune cells) and plasma that contains dissolved factors [64]. Granulocytes are the main immune cells and are crucial for phagocytosis and encapsulation of foreign agents. They neutralize these agents by releasing ROS, lysing enzymes, and humoral antimicrobial proteins [65,66]. Consequently, the number of hemocytes and the ratio of different cell types in the hemolymph are important indicators for assessing the health of the animal [67]. In our study, we observed an increase in THC and the percentage of granulocytes in scallops from the most contaminated area, Voevoda Bay, particularly among the “adult” group. This increase is typically associated with chronic infection [4,28]. We also noted a decrease in the proportion of agranulocytes and hemoblasts in adult scallops in both Voevoda Bay and Vityaz Bay. Among all specimens from Vityaz Bay, only in the “young” group was hemolymph contamination correlated directly with the THC, suggesting cell proliferation in response to infection [68]. However, in animals from Voevoda Bay, we found an inverse correlation between tissue contamination and both agranulocytes and hemoblasts across all age groups, while a direct correlation existed with granulocytes. A similar correlation pattern in younger individuals was observed in Vityaz Bay. Additionally, we found that an increase in the number of hypnospores in the hemolymph correlated with a decrease in granulation, likely due to degranulation during pathogen attacks. An increase in hemocyte numbers has also been reported in various other bivalve species infected with different pathogens. For example, *O. edulis* infected with *B. ostreae*, *C. virginica* infected with *P. marinus* and *H. nelsoni*, and *M. galloprovincialis* infected with *M. refringens* all showed an increase in hemocyte counts [69,70,71]. An increase in granulocytes was observed only in *C. gigas*, which is less susceptible to infection with *P. marinus* [72]. An increase in the number of granulocytes in the least contaminated group of mollusks may indicate their important role in resisting the pathogen.

Phagocytosis is considered one of the main defense mechanisms in bivalve mollusks. Our findings revealed that the highest levels of PA and ROS were present in the most infected individuals from Voevoda Bay and Vityaz Bay. This aligns with previous studies of La Pere and colleagues [72] and Ordás and colleagues [73]. Differential correlation analysis indicated that, in scallops from Voevoda Bay, the level of ROS decreased as the number of hypnospores increased in the gills and mantle (particularly in older specimens). Conversely, PA levels increased with the number of hypnospores in the hemolymph and digestive gland. PA increased with infection in other tissues for adult scallops, while the middle-aged group showed a decrease corresponding to contamination across all tissues, which is consistent with the results obtained previously by Anderson and colleagues [74]. In young animals from Vityaz Bay, we identified a strong positive correlation between invasion and PA, likely linked to a rise in granulocyte proportions. In adult scallops, however, we observed a contradictory trend where PA had a negative correlation with ROS, presumably due to the ineffective immune response. Research suggests that pathogens like *Perkinsus* can secrete lytic factors while within the mollusk, which suppresses the immune response of hemocytes and facilitates tissue invasion [75,76,77]. Protozoans such as *Bonamia*, *Marteilia*, and *Haplosporidium* employ similar penetration mechanisms: once ingested by hemocytes, they disrupt phagocytosis, allowing them to survive and spread throughout the host [71,78]. For example, in *C. virginica*, *P. marinus* and *H. nelsoni* have been shown to decrease phagocytosis levels [74]. Moreover, *H. nelsoni* even releases inhibitory molecules that hinder the molecular recognition process necessary for phagocytosis [74,78]. Extracellular products from *Perkinsus* are known to inhibit the production of ROS and lysozymes, as well as the bactericidal activity of hemocytes. They achieve this by neutralizing the effects of the oxidative burst using three antioxidant enzymatic systems: acid phosphatases, superoxide dismutases, and ascorbate peroxidases [28]. However, an opposing effect can also occur, particularly in eastern oysters, where various strains of *Perkinsus* lead to increased production of both ROS and Nitric Oxide [79]. Furthermore, studies have shown that *P. marinus* can reduce hemocyte motility, which is closely tied to phagocytic activity [76]. It can also release lytic factors within the mollusk, suppressing the immune response and facilitating its invasion [75]. *P. marinus* infection has been found to inhibit apoptosis in *C. virginica* [80,81]. Specifically, 45 min post-infection, all examined strains of *P. marinus* induced apoptosis in hemocytes during the first two hours. After this period, the level of hemocyte apoptosis gradually declined, likely due to *P. marinus* inhibiting the host’s apoptotic response. This ability enables the parasite to replicate for an extended period within hemocytes, using them to colonize other tissues [81].

When pathogens penetrate, there are changes in cell-mediated immunity, as well as alterations in humoral factors, including cytokines that regulate the growth and differentiation of hemocytes. The activity of pattern recognition receptors (PRRs) such as C-type lectins, Toll-like receptors (TLRs), and fibrinogen-related proteins (FREPs) also changes. Additionally, thioether-containing proteins, lipopolysaccharides, β-1,3-glucan-binding proteins, peptide-recognition proteins (PGRPs), and antimicrobial proteins show varying levels of activity in the hemolymph of mollusks [82]. For example, after infection with *P. marinus*, an increase in hemagglutinin titer was observed in the plasma of Pacific oysters [72]. The induction of hemagglutinin titer in Pacific oysters has also been reported to increase with elevated bacterial concentrations in the water [72,83]. While plasma lysozyme levels in eastern oysters with mild *P. marinus* infections showed a slight decrease, this reduction became more pronounced with higher infection levels or with *H. nelsoni* protozoa [69]. Conversely, plasma protein concentrations rose after exposure to *P. marinus* [72]. In *R. decussatus* infected with *Perkinsus*, a significant increase in the expression of a lectin specific for sialic acids was observed. This lectin appears to play a role in the recognition of parasites, complement activation, and cell migration [84]. We also noted an increase in PC in animals from the most contaminated waters, specifically Vityaz Bay and Voevoda Bay. Furthermore, the most significant increase in PC was observed in adult scallops within both water bodies, suggesting a potential role of the humoral response in resistance to the pathogen.

Among all the studied waters, Voevoda Bay and Vityaz Bay had the highest levels of contamination, particularly due to the dense stocking of individuals from aquaculture farms. In contrast, mussels from Amur Bay (Sportivnaya Gavan Bay), which is situated near the Vladivostok metropolitan area and considered a less favorable environment, exhibited lower contamination levels. The least contaminated mussels were found in Vostok Bay, which is regarded as the cleanest water body with dynamic hydrological conditions. An analysis of the immune status of scallops from all the studied areas revealed a moderate correlation with contamination levels, particularly with PA (0.37). However, a differential analysis based on tissue and age showed more notable changes in the immune systems of specimens from Voevoda Bay during *Perkinsus* infection. The most vulnerable and contaminated group consisted of 1-year-old scallops, likely due to their “poorly developed” immune systems. When the *Perkinsus* pathogen penetrates through critical barriers such as the mantle and gills, adult specimens (aged 3 years and older) exhibited an increase in PC levels in their plasma (0.53), suggesting a rapid and effective defensive response compared to the young (1-year) and middle-aged (2-year) groups. Simultaneously, mollusks displayed a reliable decrease in ROS levels (ranging from −0.25 to −0.73) and the proportion of granulocytes (from −0.40 to −0.55). However, they also showed stimulation in the proliferation and migration of hemoblasts to the lesions (from 0.23 to 0.40) in order to replenish the pool of circulating hemocytes needed to combat the pathogen. However, in the “adult” group, there was also an increase in the number of hemocytes (hemolymph—0.63, mantle—0.38, gills—0,49, digestive gland—0.31), likely due to the release of hemoblasts from reserves to eliminate the antigen. Despite this, there was a significant decrease in circulating agranulocytes (−0.61), while their numbers increased in middle-aged (0.25) and young (0.55) scallops, suggesting that agranulocytes may play a role in eliminating *Perkinsus* (there are no previous reports on this). After the pathogen penetrates the hemolymph, it appears to trigger an even greater production of proteins, not just in the adult group (0.64), which is the most stable and immunologically developed, but also in the middle-aged group (0.71). Interestingly, ROS synthesis remains low in both the adult (−0.20) and young (−0.12) groups, while it begins to increase in the middle-aged group (0.46), possibly indicating a turning point in the development of the scallops’ immune systems. Phagocytosis activity of hemocytes shows a slight increase in both the adult (0.33) and young (0.35) groups, but this is not observed in the middle-aged group. At the point of antigen penetration, the adult group maintains a higher THC (0.63), while the correlation in the other groups becomes negative (ranging from −0.15 to −0.31), suggesting a lack of available resources to combat the pathogen. In all groups, the proportion of granulocytes continues to decline (from −0.22 to −0.41) among circulating hemocytes, which may eventually lead to the depletion of protective resources. At the same time, the proportion of agranulocytes begins to rise in all groups of scallops (from 0.20 to 0.50). At the terminal stage, when *Perkinsus* penetrates the digestive gland, there is a slight increase in PC in adult scallops, along with decreases in young scallops (−0.52), as well as changes in ROS, PA, and THC levels, as well as a change in the ratio of hemocyte types (with a continuing decrease in the proportion of granulocytes and the degree of hemocyte granulation).

## 5. Conclusions

This study, which examined populations of both wild (Vostok Bay and Sportivnaya Gavan Bay) and cultured (Voevoda Bay and Vityaz Bay) *M. yessoensis*, revealed the presence of *Perkinsus* in scallops from all studied sites, each with varying ecological conditions. The highest frequency of infection was found among samples from aquaculture farms, raising concerns about the rapid spread of the pathogen in the region, especially under high-density stocking conditions. Young (1-year-old) *M. yessoensis* from the aquaculture farms at Voevoda Bay and Vityaz Bay exhibited the highest levels of contamination. In Voevoda Bay, the highest contamination levels were observed in the gills, mantle, and digestive gland in young specimens. In contrast, adult mollusks had the highest contamination levels in their shells and hemolymph, suggesting that they may act as carriers of *Perkinsus*. During the initial stages of infection, when *Perkinsus* was detected only in the hemolymph and mantle, the scallops from Voevoda Bay showed a significant increase in plasma protein concentration, with even greater increases in older specimens. This response likely represents a crucial mechanism for resisting pathogen invasion and requires further detailed investigation. When infection was detected in the mantle and gills, we noted a decrease in reactive oxygen species (ROS) and granulocytes, alongside an increase in hemoblasts and agranulocytes. This likely indicates that these types of hemocytes play a central role during infection. The levels of phagocytic activity (PA) increased only in the presence of *Perkinsus* already in the digestive gland. Therefore, the increased cellular and humoral activity observed in adult scallops (3–4 years old) contributed to a more effective resistance to the parasite.

In conclusion, the disease has a cumulative effect, and its progression is exacerbated by elevated temperatures, an immature immune system, and the presence of carriers in cage culture. Given the rapid expansion of aquaculture and the high economic value of *M. yessoensis*, it is crucial to assess not only the parasite fauna affecting scallops but also their defense mechanisms. This information can be vital in determining the outcomes of infection and in minimizing produce losses.

## Figures and Tables

**Table 1:** *Perkinsus* content in *M. yessoensis* in Peter the Great Bay, Sea of Japan (adults—3 years and older; middle-aged—2 years; young—1 year; n.a.—not applicable).

Sampling Stations (Time, Temperature, Salinity)	Age of Individuals	Number of Individuals	Contamination (Hypnospores/mL)/Percentage of Infected				
			Hemolymph	Mantle	Gills	Digestive Gland	Shell
Vostok Bay (05/2024, 8 °C, 28‰)	all	24	926 ± 579/26%	n.a.	n.a.	n.a.	n.a.
Sportivnaya Gavan Bay (05/2024, 8 °C, 32‰)	all	30	3667 ± 1470/47%	n.a.	n.a.	n.a.	n.a.
Vityaz Bay (02/2025, 0 °C, 34‰)	all	60	4963 ± 2201/43%	n.a.	n.a.	n.a.	n.a.
Vityaz Bay (05/2024, 9 °C, 34‰)	all	36	9630 ± 6034/69%	n.a.	n.a.	n.a.	n.a.
	adults	12	10,741 ± 10,712/67%	n.a.	n.a.	n.a.	n.a.
	middle-aged	12	2963 ± 1643/58%	n.a.	n.a.	n.a.	n.a.
	young	12	15,185 ± 14,536/83%	n.a.	n.a.	n.a.	n.a.
Voevoda Bay (05/2024, 9 °C, 33‰)	all	35	882,349 ± 189,697/89%	1157 ± 470/80%	4367 ± 1223/89%	334 ± 221/40%	1825 ± 536/91%
	adults	12	1,017,963 ± 329,092/100%	604 ± 355/67%	3691 ± 1956/92%	25 ± 41/8%	2457 ± 1331/83%
	middle-aged	12	763,889 ± 358,247/83%	767 ± 322/83%	4735 ± 2383/75%	150 ± 96/50%	2242 ± 578/100%
	young	11	863,636 ± 309,287/82%	2188 ± 1301/100%	4714 ± 2138/100%	873 ± 631/64%	682 ± 235/94%

**Table 2:** Correlation matrix of all parameters studied for *M. yessoensis* from Vostok Bay (Spearman’s coefficient, *p* < 0.05; significant correlations are highlighted in bold).

Vostok Bay	Protein Concentration, mg/mL	Reactive Oxygen Species, AU	Phagocytic Activity, RFU	Total Hemocyte Count, Cells/mL	Granulocytes, %	Agranulocytes, %	Hemoblasts, %	Granulocyte Granularity	Agranulocyte Granularity
Total Hemocyte Count, cells/mL	**0.50**	**−0.23**	−0.10						
Agranulocytes, %	−0.08	**−0.29**	0.19	−0.14	**−0.83**				
Hemoblasts, %	−0.12	0.10	−0.15	0.01	**−0.53**	0.02			
Granulocyte granularity	**0.25**	**−0.28**	**0.30**	0.06	0.13	0.00	**−0.31**		
Agranulocyte granularity	0.05	**−0.26**	**0.25**	−0.14	0.11	−0.01	**−0.20**	**0.55**	
Hypnospores, number/mL	−0.08	−0.16	−0.15	0.00	0.11	**−0.21**	−0.01	0.02	**−0.23**

**Table 3:** Correlation matrix of all parameters studied for *M. yessoensis* from Sportivnaya Gavan Bay (Spearman’s coefficient, *p* < 0.05; significant correlations are highlighted in bold).

Sportivnaya Gavan Bay	Protein Concentration, mg/mL	Reactive Oxygen Species, AU	Phagocytic Activity, RFU	Total Hemocyte Count, Cells/mL	Granulocytes, %	Agranulocytes, %	Hemoblasts, %	Granulocyte Granularity	Agranulocyte Granularity
Phagocytic activity, RFU	**−0.30**	**−0.54**							
Total Hemocyte Count, cells/mL	−0.03	**−0.68**	**0.62**						
Granulocytes, %	**−0.20**	**−0.20**	0.14	**0.29**					
Agranulocytes, %	**0.22**	**0.31**	**−0.21**	**−0.31**	**−0.87**				
Hypnospores, number/mL	−0.07	−0.12	0.07	−0.07	0.03	−0.06	0.01	−0.03	0.08

**Table 4:** Correlation matrix of all parameters studied for *M. yessoensis* from Voevoda Bay (Spearman’s coefficient, *p* < 0.05; significant correlations are highlighted in bold).

Voevoda Bay	Protein Concentration, mg/mL	Reactive Oxygen Species, AU	Phagocytic Activity, RFU	Total Hemocyte Count, Cells/mL	Granulocytes, %	Agranulocytes, %	Hemoblasts, %	Granulocyte Granularity	Agranulocyte Granularity
Reactive Oxygen Species, AU	**−0.38**								
Phagocytic activity, RFU	**−0.31**	0.11							
Total Hemocyte Count, cells/mL	−0.13	**0.21**	**0.23**						
Granulocytes, %	−0.14	**0.40**	−0.09	−0.08					
Agranulocytes, %	0.11	**−0.38**	0.19	0.13	**−0.94**				
Hemoblasts, %	0.19	**−0.41**	−0.15	−0.08	**−0.81**	**0.59**			
Granulocyte granularity	0.05	**0.38**	**0.21**	−0.02	0.09	−0.02	**−0.22**		
Agranulocyte granularity	**−0.20**	**0.41**	0.11	0.17	**0.25**	−0.17	**−0.32**	**0.50**	
Hypnospores, number/mL	**0.33**	0.03	0.11	0.02	−0.15	**0.20**	0.10	0.09	−0.14

**Table 5:** Correlation matrix of all parameters studied for *M. yessoensis* from Vityaz Bay (Spearman’s coefficient, *p* < 0.05; significant correlations are highlighted in bold).

Vityaz Bay	Protein Concentration, mg/mL	Reactive Oxygen Species, AU	Phagocytic Activity, RFU	Total Hemocyte Count, Cells/mL	Granulocytes, %	Agranulocytes, %	Hemoblasts, %	Granulocyte Granularity	Agranulocyte Granularity
Reactive Oxygen Species, AU	**−0.51**								
Phagocytic activity, RFU	**−0.58**	**0.49**							
Total Hemocyte Count, cells/mL	**−0.79**	**0.46**	**0.70**						
Granulocytes, %	**−0.44**	**0.35**	**0.30**	**0.61**					
Agranulocytes, %	**0.42**	**−0.37**	**−0.31**	**−0.62**	**−0.98**				
Hemoblasts, %	**0.34**	−0.08	−0.11	**−0.21**	**−0.58**	**0.46**			
Granulocyte granularity	**−0.60**	**0.62**	**0.48**	**0.61**	**0.54**	**−0.54**	−0.15		
Agranulocyte granularity	**−0.58**	**0.30**	**0.39**	**0.68**	**0.62**	**−0.61**	**−0.27**	**0.57**	
Hypnospores, number/mL	0.12	0.10	0.13	−0.06	0.02	−0.02	0.00	−0.12	**−0.20**

## Data Availability

The original contributions presented in this study are included in the article. Further inquiries can be directed to the corresponding author(s).
